# Evaluations of primary lesions by endoscopy clearly distinguishes prognosis in patients with gastric cancer who receive chemotherapy

**DOI:** 10.1371/journal.pone.0173663

**Published:** 2017-03-13

**Authors:** Tomomitsu Tahara, Tomoyuki Shibata, Masaaki Okubo, Tomohiko Kawamura, Noriyuki Horiguchi, Dai Yoshida, Takamitsu Ishizuka, Mitsuo Nagasaka, Yoshihito Nakagawa, Naoki Ohmiya

**Affiliations:** Department of Gastroenterology, Fujita Health University School of Medicine, Toyoake, Japan; Seoul National University College of Pharmacy, REPUBLIC OF KOREA

## Abstract

**Background:**

Chemotherapy may improve outcomes in gastric cancer (GC), especially for the patients with advanced stage. To explore useful predictive factor for GC performing chemotherapy, we compared the tumor responses assessed using computed tomography (CT) with endoscopy based criteria.

**Methods:**

192 GC patients performing chemotherapy were retrospectively studied. CT based response assessment was performed after 2 courses of treatment. Endoscopic evaluation according to The Japanese classification of gastric carcinoma was also performed at same period. Data were correlated with overall survival (OS) and progression-free survival (PFS).

**Results:**

Majority of the cases (n = 178, 93%) received S-1 based chemotherapy as the first line treatment. 55 (29%) and 91 (47%) cases were considered to be CT and endoscopic responders. Endoscopic responder was more clearly associated with better OS and PFS compared to CT based responder by the log-rank test (*P*<0.0001 *vs*. 0.01 and *P*<0.0001 *vs*. 0.008, respectively). The association was more striking among patients performing neoadjuvant chemotherapy (*P*<0.0001 *vs*. 0.15 and *P*<0.0001 *vs*. 0.1, respectively). Multivariate survival analysis using Cox's regression model revealed that endoscopic non-responder was the independent predictive factor, being more strongly associated with worse OS when compared to CT non-responder (hazard ratio: 4.60 *vs*. 1.77, 95% confidence interval: 2.83–7.49 *vs*.1.08–2.89, *P*<0.0001 *vs*. 0.02). More advanced T, N stage and cases who had peritoneal dissemination were significantly associated with endoscopic non-responder (all *P* values <0.01).

**Conclusion:**

Endoscopy based evaluation of primary lesions are clearly associated with prognosis in patients with GC who perform chemotherapy.

## Introduction

Gastric cancer (GC) is one of the most common malignancies worldwide, accounted for approximately 70,000 new cases and 650,000 deaths per year [1,2]. Despite advance in strategy for early detection, many patients still have advanced disease at diagnosis. Since the prognosis of patients with advanced tumor is poor [3], improved treatment outcomes in patients with advanced GC would be required to further decrease in mortality.

Chemotherapy is now recognized as the most effective treatment for patients especially with unresectable advanced or metastatic GC. To date, many clinical trials have evaluated its efficacy and the safety [4–9]. Other than unresectable cases, neoadjuvant chemotherapy can be also considered for potentially resectable cases to further improve their outcomes. Several studies have evaluated the potential usefulness of neoadjuvant chemotherapy in locally advanced GC [10–14].

Precise assessment of response to the chemotherapy would be of great importance for tailoring chemotherapy based on individual response. Correct identification of responding or non-responding patients would be particularly important to avoid toxic and ineffective chemotherapy [15–17]. Tumor response to chemotherapy is generally assessed using the Response Evaluation Criteria in Solid Tumors (RECIST) [18], but the RECIST requires the presence of a measurable lesion. In the RECIST, primary gastric tumors are regarded as non-target lesions and endoscopic diagnosis is not recommended as an objective evaluation. Since resectable GC usually doesn’t have a measurable lesion, it may be difficult to apply RECIST especially for the cases receiving neoadjuvant chemotherapy.

The Japanese Gastric Cancer Association (JGCA) developed an original method to evaluate the response of the primary gastric lesion to chemotherapy using upper gastrointestinal (GI) X-ray or endoscopy [19], but it was not widely used, mainly because of technical difficulties. However, recent study suggests the importance of evaluating the responses of primary lesions for predicting median survival times (MST) in patients with unresectable, advanced GC [20]. Other study investigating GC performing neoadjuvant chemotherapy demonstrated that an early evaluation using endoscopy is useful for predicting response and prognosis with good correlation with computed tomography (CT) and histological based response evaluation [21].

To evaluate the validity of endoscopy based response evaluation of primary lesions to chemotherapy in a GC, we investigated 192 GC including patients treated by neoadjuvant chemotherapy and chemotherapy alone to compare endoscopy based response evaluation with CT based criteria. The result demonstrated that endoscopy based response evaluation is superior to CT based evaluation for the prediction of overall survival (OS) and progression-free survival (PFS), supporting the higher response assessment validity of endoscopy based evaluation of primary lesion for predicting prognosis of GC receiving chemotherapy.

## Materials and methods

### Ethics statement

This study was approved by the Human Research Ethics Committee of the Fujita Health University School of Medicine. Each participant provided written informed consent for their clinical and laboratory data to be used and published for research purposes. The study was conducted according to the principles expressed in the Declaration of Helsinki.

### Patients, survival and response evaluation using different criteria

We retrospectively studied 192 Japanese patients with GC receiving chemotherapy from April 2003 to September 2012 in our hospital. We included all consecutive GC patients with stage II, III and IV diseases who received chemotherapy during the study period. The exclusion criteria was stage I diseases that are not usually treated by the chemotherapy.

**Fig 1:**
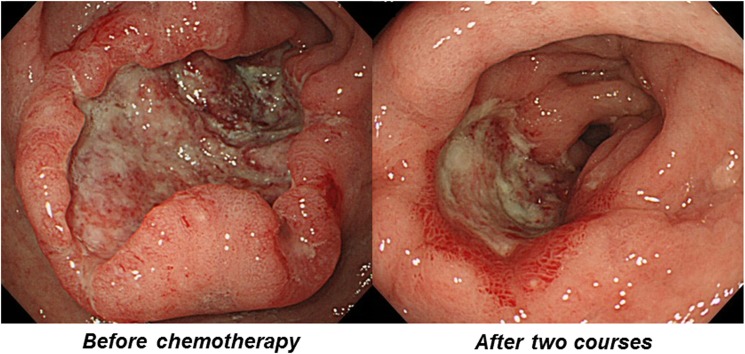
A representative case of GC considered to be endoscopic responder. An ulcerative lesion (type 3 tumor) showed distinct reduction and flattening after two courses of chemotherapy.

All GC were diagnosed histologically and were classified according to Lauren’s classification [22]. Detailed information about anatomic location, macroscopic types, depth, lymph node and other metastasis and peritoneal dissemination was obtained according to the JGCA [19]. Among the 192 patients, 78 patients were considered as operable after two courses of chemotherapy and underwent gastrectomy with a D2 lymph node dissection. For the remaining 114 cases, chemotherapy alone was used for the treatment. For the tumor response evaluation to chemotherapy, we used two different criteria (i.e. computed tomography (CT) based and endoscopy based evaluations). Using CT, response to chemotherapy was assessed after 2 courses of treatment (7 to10 weeks after initial administration, which is varying among the different regimens). If the measurable lesions were exist, response evaluation criteria in solid tumors (RECIST) [18] were applied and classified cases into complete response (CR), partial response (PR), stable disease (SD) and progressive disease (PD). CR, PR according to the RECIST was considered to be responder. If the RECIST was not applicable, responder was defined as clearly reduction of primary or metastatic lesions in the images of CT assessed by experienced physicians by the consensus manner. Upper endoscopy was also performed after 2 courses of treatment for the evaluation of primary lesion to chemotheraphy. Morphological changes of primary lesions were evaluated according to the JCGC criteria [19]. Endoscopic CR was defined as disappearance of primary gastric lesions. Measurable lesions with at least a 50% decrease in total tumor size in two dimensions and at least a 30% decrease in total tumor size in one dimension, evaluable but non measurable lesions with flattening, and diffusely infiltrating lesions with roughly at least 50% enlargement of the gastric lumen in the tumor area are all defined as endoscopic PR (Fig 1). CR and PR was considered to be responder. All assessment of endoscopic pictures was based on consensus manner from experienced two out of expert endoscopists involved in this study (TT, TS, MO, TK and TI). Those who were not considered to be responder by CT and endoscopy based evaluations were considered to be CT and upper endoscopy based non-responder, respectively.

Overall survival (OS) was defined as the time from start of initial administration of chemotherapy to the date of cancer related death. If the cancer related death has not occurred, the OS was censored on the last date the patient has known to be alive. Progression-free survival (PFS) was defined as the time of initial administration of chemotherapy to tumor progression or cancer related death. PD according to the RECIST or endoscopically visible increase in total tumor size, or reduction of the gastric lumen in the tumor area are all defined as tumor progression. Patients with no confirmation of progression or cancer related death were censored at the date of the last objective tumor assessment.

### Statistical analysis

Categorical variables among the two groups were assessed using the two-tailed Fisher's exact test. OS and PFS among the two groups were assessed using the Kaplan-Meier method and the Log rank test. Multivariate survival analysis using Cox's regression model was also performed for calculating hazard ratio (HR) and 95% confidence interval (CI) with adjustment of clinicopathological factors. A P value less than 0.05 was considered as statistically significant.

## Results

### Gastric cancer patients and their treatment

Clinic-pathological characteristics of subjects and information about treatment are shown in Table 1 and Table 2. Majority of the cases (n = 178, 93%) received S-1 based chemotherapy (S-1+CDDP, S-1+DTX, S-1 alone, DTX+CDDP+S-1) as the first line treatment. Regarding numbers of regimens used during the treatment, 134 (69.8%) and 24 (12.5%) patients received first line and second line treatment, while 33 (17.2) patients received third line (or more) treatment. After two courses of treatment, CT and endoscopy based response evaluation was performed for 190 and all patients, respectively. 55 (29%) and 91 (47%) cases were considered to be CT and endoscopic responders (Table 2). 78 patients were considered to be operable after two courses of chemotherapy and underwent gastrectomy with a D2 lymph node dissection but during surgery, distant metastatic lesions were found in 13 cases (data not shown).

**Table 1:** Clinic-pathological features of gastric cancers.

Variables n (%)	
*Total number of patients*	192
*Median age*	68 (30–90)
*Gender*	
Female	55 (28.6)
Male	137 (71.4)
*Location*	
Upper	53 (27.6)
Middle	88 (45.8)
Lower	51 (26.6)
*Histology*	
Intestinal	66 (34.4)
Diffuse	92 (47.9)
Mixed	31 (16.1)
*Morphology*	
Type1	7 (3.65)
Type2	50 (26.0)
Type3	106 (55.2)
Type4	29 (15.1)
Other	
*Staging*	
II	35 (18.2)
III	53 (27.6)
IV	104 (54.2)
*Depth*	
T2	17 (8.6)
T3	19 (9.9)
T4	156 (81.3)
*Lymphnode metastasis*	
N0	51 (26.6)
N1	30 (15.6)
N2	41 (21.4)
N3	70 (36.5)
*Distant metastasis*	
Peritoneal dissemination	70 (36.5)
Liver metastasis	35 (18.2)
Other metastasis	28 (14.6)

**Table 2:** Information about treatment of gastric cancers.

Variables n (%)	
*Treatment*	
Chemotherapy alone	114 (59.3)
Neoadjuvant chemotherapy	78 (40.6)
*Agent*	
S-1+CDDP	133 (69.3)
S-1+DTX	23 (12.0)
S-1	18 (9.4)
PTX	6 (3.1)
DTX+CDDP+S-1	4 (2.1)
Others	8 (4.2)
*Response to chemotherapy (CT)$*	
Responder	55 (29.0)
Non-responder	135 (71.0)
*Response to chemotherapy (endoscopy)*	
Responder	91 (47.4)
Non-responder	101 (52.6)
*Number of regimens for chemotherapy #*	
First line	134 (69.8)
Second line	24 (12.5)
Third line or more	33 (17.2)

$ Information was not available for two patients.

# Information was not available for one patient.

### Superiority of endoscopy based response evaluation than CT

**Fig 2:**
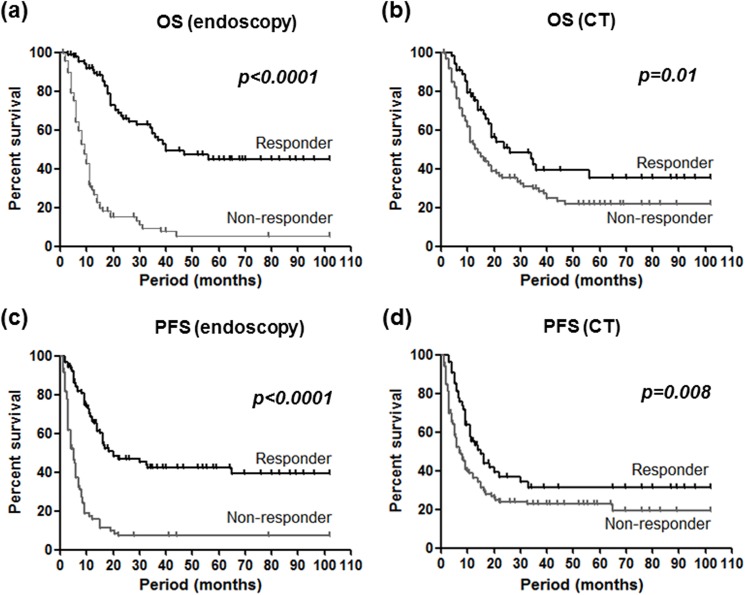
Overall survival (OS, upper) and progression-free survival (PFS, lower) and endoscopy (left) and CT (right) based response evaluations. Different two groups was assessed using the Kaplan-Meier method and the Log rank test.

OS and PFS could be assessed in all and 191 cases, respectively. The median OS and median PFS were 12 and 7.5 months, respectively. In overall, endoscopic responder was more clearly associated with better OS and PFS compared to CT responder by the log-rank test (*P*<0.0001 *vs*. 0.01 and *P*<0.0001 *vs*. 0.008, respectively, Fig 2).

**Fig 3:**
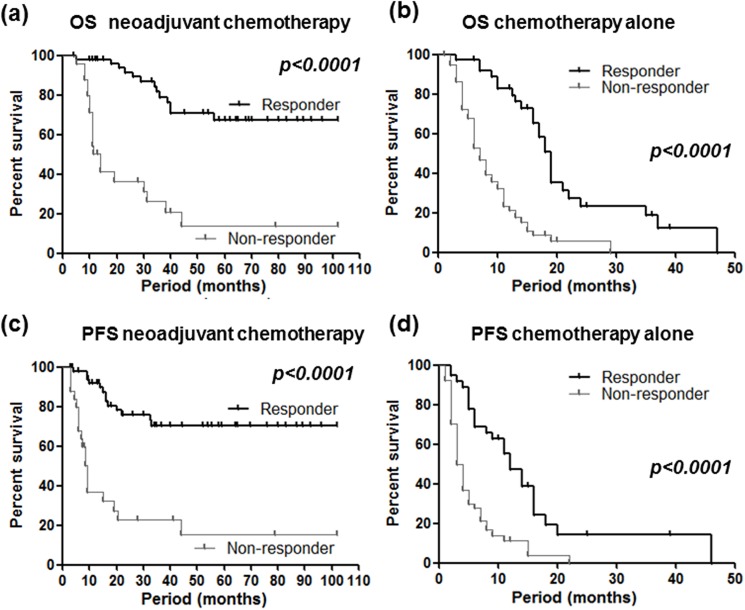
Overall survival (OS, upper) and progression-free survival (PFS, lower) in relation to the endoscopy based response evaluation. Left, patients performing neoadjuvant chemotherapy; right, patients performing chemotherapy alone; Different two groups was assessed using the Kaplan-Meier method and the Log rank test.

**Fig 4:**
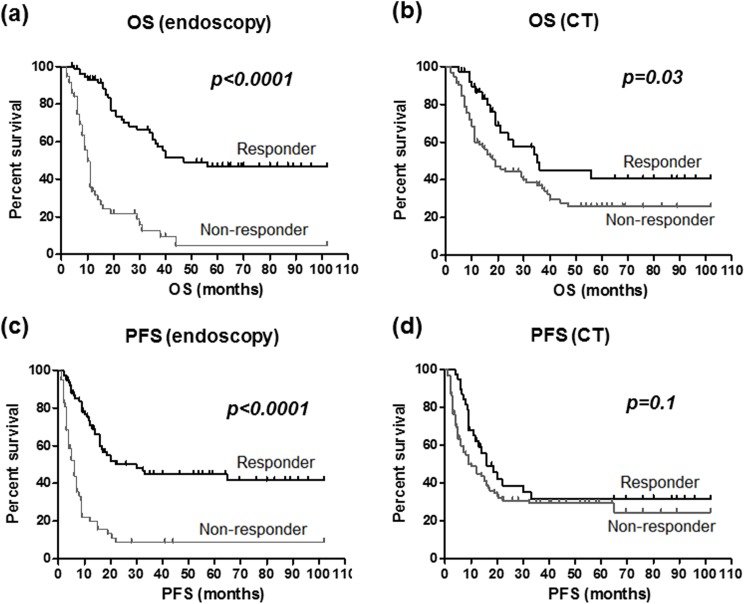
Overall survival (OS, upper) and progression-free survival (PFS, lower) and endoscopy (left) and CT (right) based response evaluations in cases who received S1 plus CDDP. Different two groups was assessed using the Kaplan-Meier method and the Log rank test.

**Fig 5:**
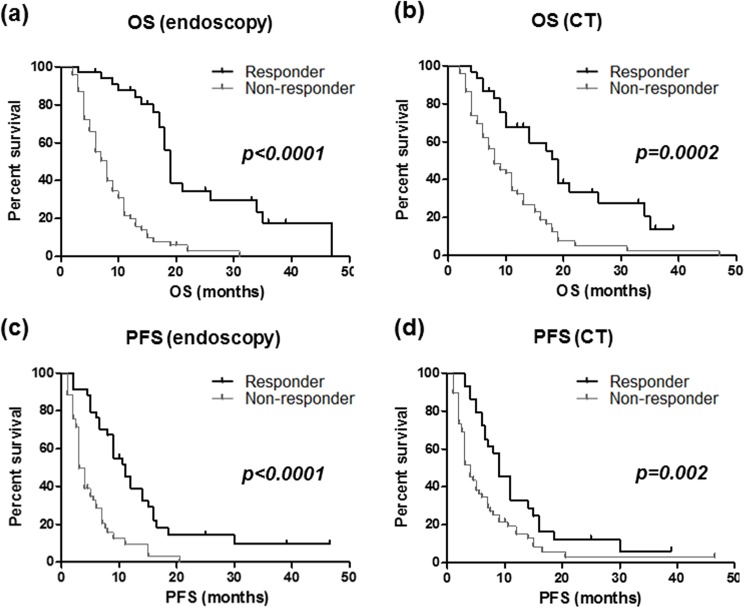
Overall survival (OS, upper) and progression-free survival (PFS, lower) and endoscopy (left) and CT (right) based response evaluations in stage IV cases that have peritoneal dissemination or distant metastasis. Different two groups was assessed using the Kaplan-Meier method and the Log rank test.

When cases were divided into two groups (i.e. neoadjuvant chemotherapy and chemotherapy alone groups), endoscopy based evaluation also clearly predicted both OS and PFS in both groups (all *P* values <0.0001, Fig 3), and the superiority of endoscopic response evaluation than CT for prediction of OS and PFS was also confirmed in these groups (data not shown). We also investigated association between endoscopy or CT based response evaluations and prognosis of GC in cases who received S1 plus CDDP, which is most frequently chosen in GC [7]. The result also demonstrated a superiority of endoscopic response evaluation compared to the CT for both OS and PFS (Fig 4). On the other hand, both the endoscopic and CT based response evaluations similarly predicted OS and PFS in stage IV cases that have peritoneal dissemination or distant metastasis (Fig 5).

**Fig 6:**
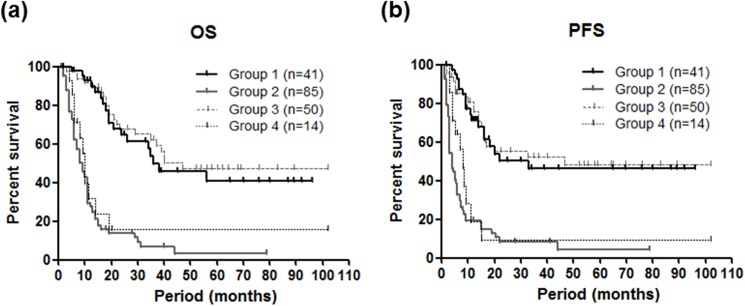
Overall survival (OS, left) and progression-free survival (PFS, right) among four groups divided according to the endoscopy and CT based response evaluations. Group 1, responder by both CT and endoscopy; group 2, non-responder by both CT and endoscopy; group 3, non-responder by CT but responder by endoscopy; group 4, responder by CT but no-responder by endoscopy. Different groups was assessed using the Kaplan-Meier method and the Log rank test.

To further confirm superiority of endoscopic response evaluation to chemotherapy to predict OS and PFS, we divided cases into four groups (i.e. group 1, responder by both CT and endoscopy; group 2, non-responder by both CT and endoscopy; group 3, non-responder by CT but responder by endoscopy; group 4, responder by CT but no-responder by endoscopy;) and compared OS and PFS. The result demonstrated that group 1 and 3 presented better OS and PFS and the survival curves of these groups were quite similar. On the other hand, group 2 and 4 presented worse OS and PFS and the survival curves of these groups were also quite similar. Since the survival curves of group 1 and 3, as well as group 2 and 4 presented similar patterns with no significant differences by the log-rank test (*p*>0.4), higher response assessment validity of endoscopy based evaluation was suggested compared to the CT (Fig 6).

### Multivariate survival analysis

To evaluate independent prognostic factors that are associated with OS in GC who receive chemotherapy, multivariate survival analysis using Cox's regression model was performed. For this analysis, all clinic-pathological factors including gender, age, anatomic location, macroscopic and histologic types and depth, information about metastasis and staging and response against treatment were included. This analysis demonstrated that endoscopic non-responder was the independent predictive factor, being more strongly associated with worse OS when compared to CT non-responder (*P*<0.0001 *vs*. 0.02). Chemotherapy alone and higher stage were also showing up as the independent predictive factor for worse OS (*P* = 0.001 and 0.003) (Table 3).

**Table 3:** Multivariate survival analysis using Cox's regression model for adjustment of clinicopathological factors.

Variables	HR (95%CI)	p value
CT (non-responder)	1.77 (1.08–2.89)	0.02
Endoscopy (non-responder)	4.60 (2.83–7.49)	<0.0001
Treatment (Chemotherapy alone)	2.78 (1.49–5.17)	0.001
Advanced stage	2.44 (1.367–4.38)	0.003

### Association between endoscopy based response and various clinic-pathological characteristics of GC

Finally, we investigated the association between endoscopy based response and various clinic-pathological characteristics of GC. Endoscopic non-responder was significantly associated with mixed or diffuse type histopathology (*P* = 0.03), higher T and N factors and staging (*P*<0.0001, 0.008, <0.0001), peritoneal dissemination (*P* = 0.0003) and chemotherapy alone group (*P*<0.0001) (Table 4).

**Table 4:** Association between clinicopatological characteristics of gastric cancer and response to chemotherapy based on endoscopy.

Variables (%)	Responder	Non-responder	*p* value
*Total number*	91 (47.4)	101 (52.6)	NS
*Age*			
75<	75 (39.1)	79 (41.1)	
75 = <	16 (8.3)	22 (11.5)	NS
*Gender*			
Female	25 (13.0)	30 (15.6)	
Male	66 (34.4)	71 (37.0)	NS
*Location*			
C	23 (12.0)	30 (15.6)	
M	45 (23.4)	43 (22.4)	
A	23 (12.0)	28 (14.6)	NS
*Morphology*			
Type1	3 (1.6)	4 (2.1)	
Type2	30 (15.6)	20 (10.4)	
Type3	46 (24.0)	60 (31.3)	
Type4	12 (6.3)	17 (8.9)	NS
*Histology$*			
Intestinal	38 (19.8)	28 (14.6)	
Diffuse	44 (22.9)	48 (25.0)	
Mixed	9 (4.7)	22 (11.5)	0.03
*Depth*			
<T3	28 (14.6)	8 (4.2)	
T4	63 (32.8)	93 (48.4)	<0.0001
*Lymphnode metastasis*			
N0-1	46 (24.0)	35 (18.2)	
N2-3	45 (23.4)	66 (34.4)	0.008
*Distant metastasis*			
Peritoneal dissemination	21 (10.9)	49 (25.5)	0.0003
Liver metastasis	13 (6.8)	22 (11.5)	NS
Other metastasis	9 (4.7)	19 (9.9)	0.08
*Staging*			
II-III	57 (29.7)	31 (16.1)	
IV	34 (17.7)	70 (36.5)	<0.0001
*Treatment*			
Chemotherapy alone	38 (19.8)	76 (39.6)	
Neoadjuvant chemotherapy	53 (27.6)	25 (13.0)	<0.0001

$: Information was not avairable for three patients.

## Discussion

The current study demonstrated that response evaluation of by endoscopy has a higher validity for predicting OS and PFS in GC receiving chemotherapy.

RECIST is the most widely accepted criteria for evaluating response to chemotherapy but it assesses only measurable metastatic lesions and primary gastric tumors are regarded as non-target lesions [18]. Endoscopic diagnosis of the primary tumor is not widely established possibly due to technical difficulties and invasiveness. However, the JGCA original criteria aimed to evaluate the response of the primary gastric lesion, which is also applicable for non-measurable lesions [19]. Several result proposed the importance of evaluating primary lesions has been suggested in both unresectable cases [23] and locally advanced cases receiving neoadjuvant chemotherapy [21]. Our result was also in line with these studies for supporting the clinical significance of evaluating primary tumors.

Park et al. demonstrated a strong prognostic influence of endoscopy based evaluations on metastatic GC, in which endoscopic and CT based response were equally associated with survival but with low correlation [23]. Other study has shown higher prognostic accuracy of endoscopy based response with good correlation with CT in cases performing neoadjuvant chemotherapy [21]. Our result suggested that endoscopy based response evaluation was superior to CT based evaluation for the prediction of OS and PFS. Multivariate survival analysis confirmed that endoscopic non-responder was stronger predictor of worse OS than CT non-responder. The correlation of endoscopy and CT in our study was fair but not excellent (k coefficient value = 0.31, data not shown). The difference seen in our result might be due to patient constitution and different time points chosen for response evaluation. The cases evaluated by Park et al. were mostly more advanced cases and endoscopic diagnosis was performed after three cycles of chemotherapy, while CT response was defined as the best response of all assessment during three cycles [23]. Other study investigated smaller case numbers of locally advanced GC and the response assessment was earlier than our study [21]. When cases were divided into four categories based on the different response evaluations, survival curves of cases in cases showing different response between endoscopy and CT suggest that result endoscopy was more accurate for the prediction of OS and PFS (Fig 3). The superiority of endoscopy based response evaluation seemed to be more striking among cases receiving neoadjuvant chemotherapy (Fig 4), while both the endoscopic and CT based response evaluations similarly predicted OS and PFS in stage IV cases that have peritoneal dissemination or distant metastasis (Fig 6), suggesting that endoscopy based evaluation in after two courses is higher validity to predict response than CT especially in cases receiving neoadjuvant chemotherapy. Since resectable GC usually doesn’t have a measurable lesion, it would be difficult to apply RECIST especially for the cases receiving neoadjuvant chemotherapy. In such cases, clearly reduction of primary or metastatic lesions in the images of CT was considered to be responder. However, CT based evaluation might be difficult to assess the response and endoscopy will be more suitable especially for locally GC receiving neoadjuvant chemotherapy. Since precise assessment of response to the chemotherapy would be of great interest for tailoring chemotherapy based on individual response. Endoscopy based response assessment seemed to be useful for identification of responding or non-responding patients to avoid toxic and ineffective chemotherapy [15–17]. In GC patients receiving neoadjuvant chemotherapy, 7.5% (4/53) endoscopic and 12% (3/25) CT responders found distant metastatic lesions during surgery, respectively. Whether endoscopic based response assessment would precisely exclude possibility of distant metastasis in GC receiving neoadjuvant chemotherapy need to be evaluated in a larger cohort.

One limitation of endoscopic diagnosis is technical difficulty. In this study, all assessment of endoscopic pictures was based on consensus manner from experienced two endoscopists, therefore, its applicability for all the physician need to be evaluated. Regarding the clinic-pathological characteristics of GC and endoscopy based response, non-responder was characterized as more advanced or aggressive phenotypes including mixed or diffuse type histopathology, higher T and N factors and staging, peritoneal dissemination, and cases performing chemotherapy alone. Based on this result, it seemed that simply the advanced or aggressive phenotypes might be associated with endoscopic non-responders.

## Abbreviations

GC: gastric cancer
CT: computed tomography
OS: overall survival
PFS: progression-free survival
